# Treatment of Exposed Dental Pulp

**Published:** 1850-10

**Authors:** H. K. Nutz

**Affiliations:** Dental Surgeon, Philadelphia.


### ARTICLE IX.

*Treatment of Exposed Dental Pulp.* By H. K. Nutz, Dental Surgeon, Philadelphia.

Dr. C. A. Harris:

*Dear Sir:*—Much has been published of late respecting the treatment of the dental pulp. It appears that the arsenical preparations are held pre-eminent by many dental practitioners; but, sir, experience has taught me that very little permanent good has been obtained from the application of arsenic: on the contrary, it has been productive of much injury, for, in the majority of cases, it gives rise, sooner or later, to inflammation of the alveolo-dental periosteum, thereby producing the most intense pain, often followed by exostosis, and most frequently the loss of the tooth, besides generally causing nervous irritation of the general system.

In my opinion, it is doubtful practice to destroy the pulp in preparing teeth previous to filling; therefore it should be avoided as much as possible, inasmuch as it destroys the vitality of the tooth to a very great extent, and frequently causes their entire death.

Experience has led me to believe that the nervous pulps of the teeth are endowed with recuperative power, so that when wounded, cicatrization will take place, provided it is not followed by suppurative inflammation, and this I have learned from observation is not an inevitable result.

The following is the manner in which I have treated exposed pulps during the last twelve months, and with entire success. In the first place, I remove all loose matter from the cavity, then apply cold water three or four times in rapid succession, by means of a syringe with a curved tube. This enables me to benumb, in a measure, the pulp, and whilst in this state I cut off, with a suitable excavator, that portion of the nerve which is near the orifice of the cavity, without producing excessive pain. I then repeat the water, and continue its application daily until I have deprived the pulp of much of its sensibility, taking care to apply a lock of cotton over the pulp, so as to protect it; this should be done after each injection. One week is sufficient to accomplish the healing.

I then proceed to fill the tooth, by first cutting a strip of gold foil, and folding it in such a manner as to enable me to lay a floor over the nerve-cavity, and then fill and condense the gold in the usual manner.

